# Can technology be good for health? Investigating health-promoting strategies in the private sector

**DOI:** 10.3389/fpubh.2024.1395422

**Published:** 2024-09-25

**Authors:** Brittany E. Sigler, Keshia M. Pollack Porter, Lindsay Thompson, Sara Singer, Darrell J. Gaskin

**Affiliations:** ^1^Bloomberg School of Public Health, Johns Hopkins University, Baltimore, MD, United States; ^2^Carey Business School, Johns Hopkins University, Baltimore, MD, United States; ^3^School of Medicine, Stanford University, Stanford, CA, United States; ^4^Graduate School of Business, Stanford University, Stanford, CA, United States

**Keywords:** consumer technology, private sector, health impact, business strategy, commercial determinants of health

## Abstract

**Introduction:**

This research investigates what might motivate tech companies and impact-driven investors to adopt a health-promoting strategy in their product development and capital allocation strategies.

**Methods:**

Participants were recruited for semi-structured interviews through purposive and snowball sampling. From 83 outreach attempts, thematic saturation required 19 completed interviews out of the 46 consumer technology executives and impact-focused investors who responded. Interviews were analyzed using grounded theory-based content analysis.

**Results:**

Seven coding categories resulted from inductive coding, with 83 sub-codes. The primary themes were: product-based health impact is magnified when matched to user demographics (making an equity mindset important); stakeholders are eager for reliable health metrics, especially those that hold across industry verticals; when capturing health impact, it is critical to include positive (i.e., economically beneficial) externalities. These results allowed for the creation of a logic model with a recommended theory of change for the private sector to develop health strategy.

**Discussion:**

Intentional integration of impact strategy with business priorities will allow teams to design products that promote health, driving buy-in and resource allocation while attracting investment and double returns. For policymakers, it is clear that tech policy and regulation for corporate reporting need to keep pace. These findings are limited by the purposive recruitment of participants, introducing potential bias and risk to generalizability.

## Introduction

Rideshare services make cars more convenient than public transit, streaming platforms make it easy to ignore signs of fatigue with auto-play, food delivery apps make fast food even faster, and social media platforms remove the intimacy of personal relationships. Since 85% of the population are smartphone users (96% of those 18–49), there is staggering potential for these products to influence the environment in which individuals eat, sleep, move, and interact (1). These are the same behaviors purported to drive the ever-increasing incidence of chronic disease, from obesity and diabetes to heart disease, depression, and anxiety (2–4). What if these products were instead designed to produce health-promoting behaviors? What if the healthy choice was the easy choice?

While there is significant literature on the use of technology in healthcare (5–8), there is minimal available evidence on the opportunity to leverage the technology that individuals use in their daily lives to improve their health (9). Where such evidence does exist, authors acknowledge the promise of technology redesign but identify tradeoffs in potential design approaches (10, 11). For example, health supportive technologies have been developed for food delivery and video streaming platforms, but the filters that highlight healthy food are not prioritized by default and the wellness reminders to pause viewing place the onus for action on the individual viewer.

**Figure 1:**
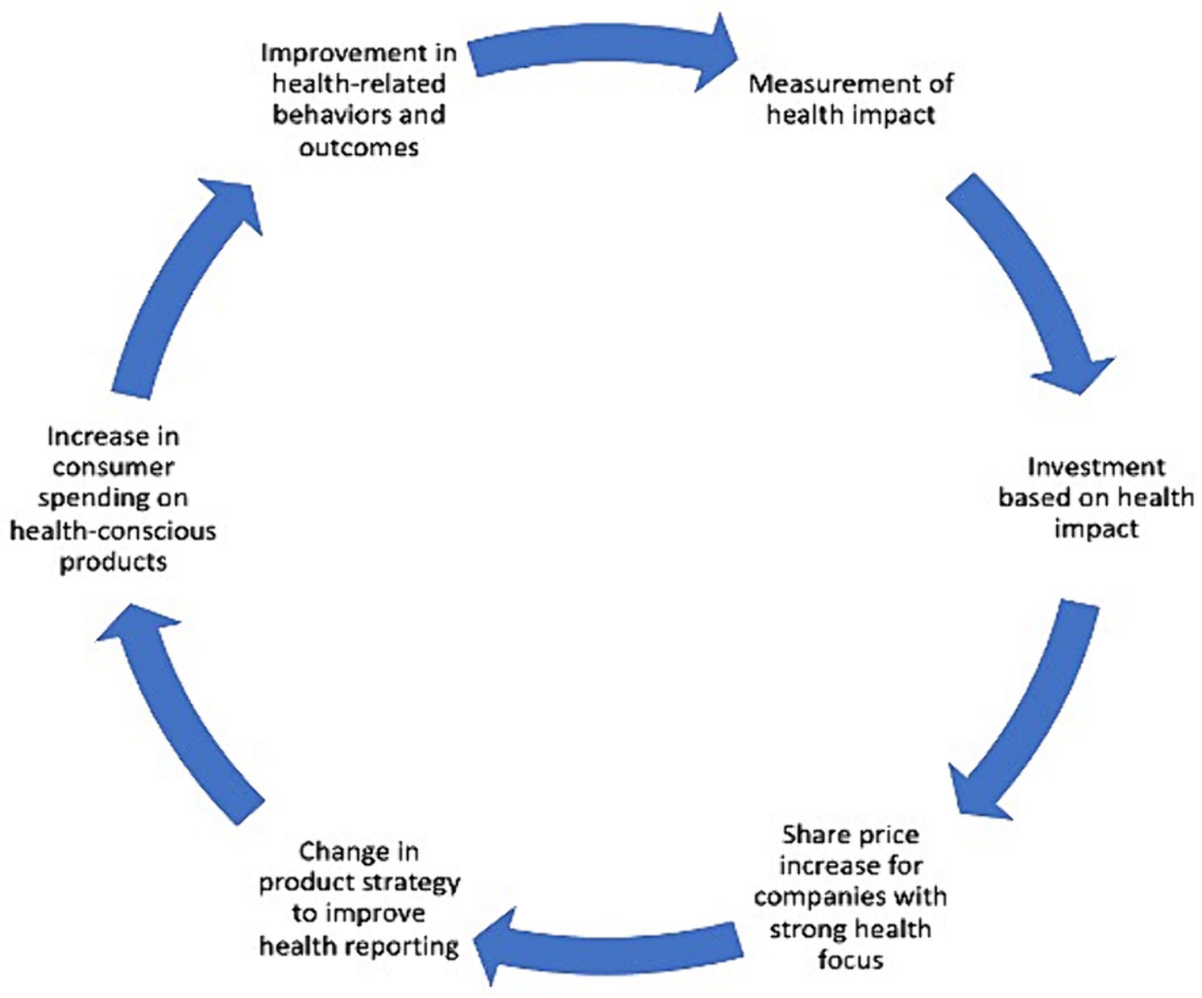
Conceptual model: health in all companies (32, 50). Adapted from Quelch & Boudreau’s (32) Culture of Health Framework and Finlay’s (50) framework for responsible investing.

The private sector (technology companies here specifically) has an opportunity to use its outsized influence to meaningfully contribute to public health. To date, there is public concern about the negative health impacts of digital products on consumers, as seen with the Congressional hearings on the influence of Facebook and Instagram on adolescent mental health (12). Likewise, existing analyses of the commercial determinants of health tend to demonstrate only negative impact of consumer products, or place the onus on individuals to change behaviors (13–17). However, private companies can exert an exponential positive influence on their users once they understand that considering the health impact of their products: (1) can produce dual returns, both social and financial (18–20), (2) can be integrated into existing strategy, and (3) reporting on these efforts can spur a cycle of investment and consumer purchasing (depicted in Figure 1). In this paper, we aim to surface recommendations for how companies can integrate health into their social impact strategy, which can likewise act as indicators for impact-driven investors (i.e., whose investment philosophies prioritize both social and financial returns).

Creating shared value by combining efforts to maximize profits and benefit society is relatively new, but gaining traction, though most discussion has focused on social and environmental benefit (21–24). Market patterns suggest eagerness for corporations to pay attention to health. Consumers want more healthy choices (22, 25, 26). Investors prefer socially responsible investing (27, 28). In turn, corporate social impact efforts reflect these trends (whether marketing around social issues, environmentally-conscious supply chain modifications, and/or emphasis on diverse hiring) (20, 29–31). If the private sector fails to take accountability for the health impact of their products on consumers, they will be at a competitive disadvantage. This paper introduces to this discourse the viability of such health-positive product development.

All companies can create a “culture of health,” in which “health effects are consistently discussed and considered in everyday corporate decision-making.” (32) Just as Health in All Policies has yielded the consideration of health externalities in non-health policies (33), the same can be achieved for products developed outside the healthcare sector, i.e., what this paper is calling “Health in All Companies.”

The private sector can help promote sustainable health through increased capital allocation to the companies that exert daily influence over lifestyle behaviors. By determining the opportunities for private sector companies to change their organizational behavior to positively influence the health of their consumers, we can motivate and measure health-promoting actions. This paper will explore the following questions:

1. Are key stakeholders already considering health in their social impact strategy? Are they investing in health outcomes, whether financially or through their prioritization?
2. How might we motivate tech companies to integrate health-promoting goals, and associated measurement, in their impact strategy and product development? What are the barriers?

## Methods

### Design, setting, and participants

We identified senior leaders (i.e., director, head, lead, or vice-president titles) in the United States through purposive and snowball sampling for semi-structured interviews. The sample was limited to U.S.-based companies due to constraints in time and access to participants, allowing for a manageable and focused research process with context-specific findings. Leveraging the Building H Index, which evaluates products based on five health-related behaviors and their consequences (34), we listed companies most influential on the same five lifestyle behaviors: how people eat, move, sleep, socialize, and spend time outside. Our sample included streaming platforms, rideshare apps, social media platforms, and food delivery companies. These companies were selected for their impact, respectively, on sleep habits, physical activity and daily movement, social interaction, and eating habits. We cross-referenced the list with exchange-traded funds focused on innovative technologies to identify gaps (35, 36). We used company “people” pages on LinkedIn to identify relevant prospects using the search terms “social impact,” “corporate social responsibility OR CSR,” “ESG,” “policy,” and “responsible innovation.” We used RocketReach for cold outreach, searching by LinkedIn URL for corporate emails. Where emails were unavailable, we sent direct messages on LinkedIn.

We identified impact-driven investors using an analogous methodology. We drafted an initial list of potential investment firms by consulting The Banker’s Investment Banking Awards for sustainable investing categories, and the LinkedIn search terms “impact investing,” “ESG,” and “socially responsible investing.” We recruited investors with a social impact focus, and if possible, a technology-centric investment thesis, or ones that worked at large banks, venture capital firms, and private equity firms.

Where there was a dearth of respondents, or, a particular company of interest, we sourced interviewees through first and second-degree networking.

After conducting outreach to 83 individuals, 37 had no response after three attempts, eight declined, four offered referrals to colleagues in their place, and four deferred participation. We made outreach attempts at approximately 2 week intervals. Those who declined explained their decision, citing either lack of time or expertise. A single interviewer conducted interviews with 19 stakeholders, nine investors, nine company employees, and one individual currently in an investor role who was previously a company employee. Those that did not respond were either very senior, or poorly targeted, with both resulting in a lack of interest or time to participate. Not all recruitment occurred on the same timeline as after 10 interviews, responses were skewed toward the investor stakeholder group. As a result, additional interviews were determined to be required with company employees to achieve thematic saturation. Ten prospective interviewees were not interviewed after having achieved thematic saturation: these were individuals who responded to initial outreach, or who were referred through past interviewees, but had not yet consented to participate. Recruitment efforts ceased as interviews were no longer yielding new insights, indicating readiness for analysis. Examples of titles of those interviewed include:

- At companies: Director of Social Impact; Senior Lead, Global Social Impact; Head of Policy Partnerships + Social Impact; Product Director, Health & Wellness Platform; Policy Director.
- Among investors: Head of Mission Investments; Director, Impact Investments; VP, Impact Investing; SVP, ESG Program Executive; Partner, Impact Investing & ESG.

The technology employees interviewed maintain accountability for considering the impact of their companies’ products on their consumers, typically under the umbrella of social issues. The investors all have impact-based theses: that is, they allocate the capital under their jurisdiction based not only on commercial returns but impact as well. These interviewees have a responsibility to consider the impact of their products and investments, and as such, are not your average employees or investors—they are intentionally leaders in the impact space.

This research project underwent review by the Johns Hopkins University Bloomberg School of Public Health Institutional Review Board (IRB), and was deemed exempt as it does not involve human subjects or poses minimal risk to participants.

### Data collection and analysis

**Figure 2:**
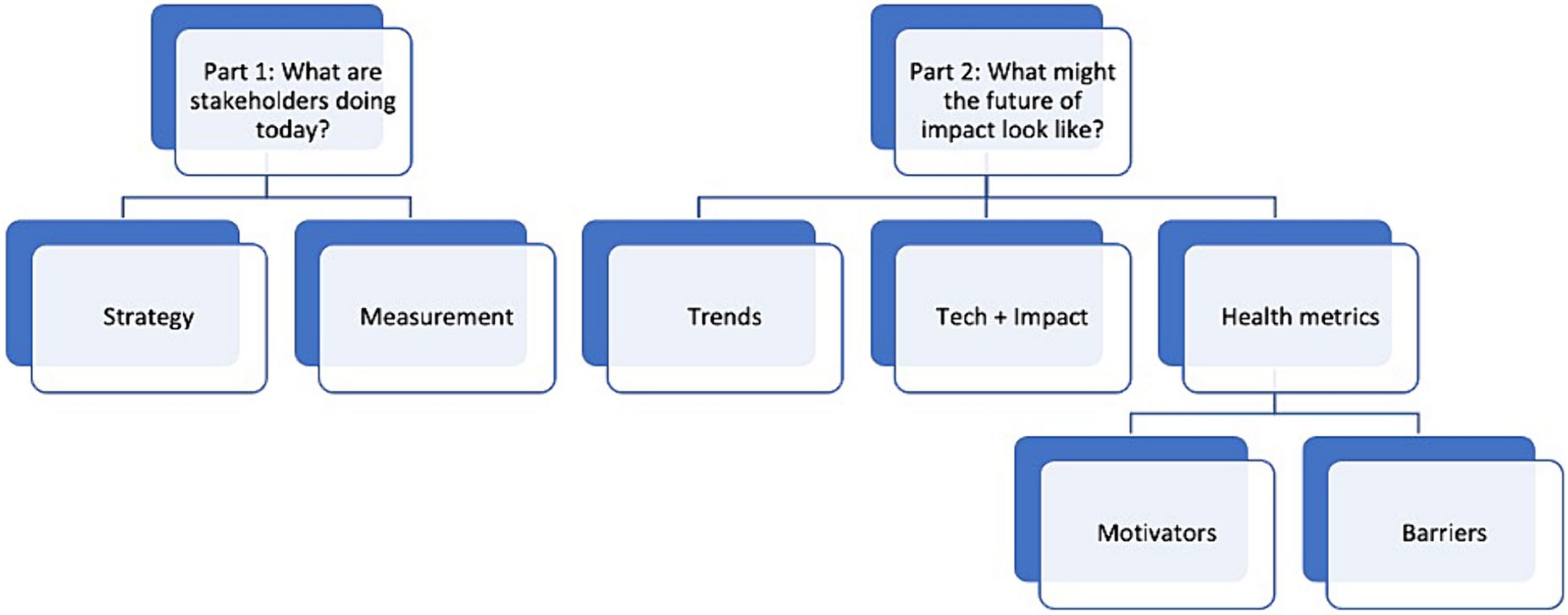
Interview domains.

Data collection occurred between October 2021 and January 2022. We intended the interview guide to elicit participants’ in-depth perspectives on the following domains: current social impact strategy, its measurement, whether and how health might influence the latter, the future of impact for tech and health, and motivators and barriers to implementing a health-promoting strategy (full interview guide in Appendix 1). Figure 2 visualizes the seven categories pursued and used for analysis.

Interviews averaged 45 min and were audio-recorded and transcribed directly through the Zoom video conferencing software used to conduct the sessions. We conducted interviews until we achieved thematic saturation, determined based on ongoing data review up to the point at which no new themes, patterns, or insights emerged from the data being collected.

Supported by NVivo (version 12.7.0), we used a grounded theory approach based in content analysis to interpret the interview transcripts. Grounded theory is most appropriate for research that seeks to discover something new, grounded in the data collected from those who experience the process—here, the intention is to develop a theory regarding the motivators and path to implementation for stakeholders integrating health into their impact strategy (37, 38). Content analysis entailed creating deductive codes based on the questions in the interview guide, yielding the overarching coding categories (blue in the codebook, see Appendix 2). This was followed by inductive coding under those categories by reading and re-reading the transcripts: allowing for the quantification and analysis of the presence and relationship of repeated words, themes, and concepts among interviewees to determine emergent themes and patterns (39, 40).

After coding was complete, a second coder performed a validity check for 25% of transcripts (41). The second coder and first author compared codes and crosswalked the transcripts to confirm face validity.

## Results

The sample yielded findings from nine technology companies representing five different sectors (software, communications, entertainment, e-commerce, food). Seven high-level coding categories resulted from the inductive coding process (Table 1), with 83 subcodes discussed by both stakeholder groups (full list in Appendix 2). Through the coding process, we merged several categories (i.e., we merged health in technology with social impact in technology into a larger called impact in technology), as interviewees tended to describe their approach to investing as one that encompassed impact more broadly, instead of differentiating between health and social impact. Another category emerged, which we called “process for metric development.” We used a generic label rather than one focused on health as the identification of health metrics was rarely discussed without attention to the successes and failures of the process itself for developing said metrics. Other thematic categories (i.e., defining health metrics, current frameworks, measurement of success, frameworks in future) became subcodes, given less robust or infrequent discussion, suggesting that these subcodes may have been less important to stakeholders, or at least topics about which the interviewees had less decisive opinions. Some interview prompts (explaining barriers) became high-level categories because of their importance in answering the guiding questions. Table 2 shares representative subcodes to provide more color on the types of ideas captured. Note each interviewee was numbered as they were de-identified as part of the analysis.

**Table 1:** Coding scheme × number of excerpts.

Codes	Company	Investor	Total
Development of social impact strategy	70	52	122
Current use of measurement	101	91	192
What’s next (future)	42	45	87
Impact in technology (combines health + social impact in technology categories, includes example health metrics)	186	78	264
Process for metric development (emergent theme informing *Discussion*)	34	20	54
Motivators for health metrics	100	63	163
Barriers for health metrics	44	27	71
Total	577	376	953

**Table 2:** Coding breakdown.

Codes	# Subcodes	Example subcodes	Sample quote
Development of social impact strategy	8	Business-integrated impact strategy, cross-functional collaboration and alignment	“We do not have a clear overarching strategy connecting all of this work together, we all work really closely together, we are all aware of each other’s work.” - Company #4
Current use of measurement	9	Current use of frameworks, measurement as prioritization	“The frameworks do not, on the ESG side or risk frameworks, so they actually do not have… there’s not a formal toolkit for unlocking value, and so we had to create it ourselves. Frankly, we did not want to.” -Investor #5
What’s next (future)	10	Interplay between privacy and social impact, frameworks in future	“I think what’s really important for companies who maybe do not have as much of a strong tie to the impact piece is taking some time to better understand what’s the why of what they are doing. And you know really getting down to that like pie in the sky dream.” - Company #2
Impact in technology	11	Equitable development of products, health in all companies	“It is important to ask these questions and to say okay well if there is potentially harm that comes from using this product. How do we ensure as individuals as families and communities and just companies that we are doing the right thing for people in the long run, because in some ways, all of this is unknown… you know we are the first generation to really leverage technology in such a way that it is a 24/7 device that we have with us at all times.” - Company #1
Process for metric development	9	Process as organizing principle, process measures as proxies	“We’re really clear about what is impactful, and how we define true success and that theory of change is the true success. The evaluation of this work is years to come, as I’m sure you know. We’re starting going deeper on evaluation now. We expect to not release the true impact of these investments for another five to seven, and maybe even 10 years because it takes that long to see real change.” - Investor #9
Motivators for health metrics	18	Influence of regulatory bodies, marketability of positive impact	“I wonder if the government can incentivize these kinds of data collection efforts that could be a fascinating unlocking of this.” -Investor #4
Barriers for health metrics	12	Burden of reporting unique metrics, costly to stand up programs	“There might be certain investment strategies that ultimately would be great for a company over a three to 5 year period and might be great for overall society. But. Every incentive says go for the money today.” - Investor #1

### Social impact strategy today

Social impact strategy guided investment, product development, or partnership decisions. Across stakeholders, strategy needed to be integrated into the business mission: allocating capital toward maximizing the opportunity for double returns, both commercial and impact. For investors, this is true regardless of sector: impact-driven investors want to find where they can best foster good with their capital. If a company chooses to tackle social issues without ties to an existing product and in-house expertise, the effort is unlikely to be successful—whether due to lack of cross-organization buy-in, sustained funds, or available capacity as this initiative would be apart from business-as-usual expectations. For example, it makes sense that a meal kit-delivery company would take on the issue of food insecurity, or for an e-commerce company to explore how they might impact financial inclusion. All companies have an opportunity to identify the social issues that most resonate with their consumers, and them as a company:

*Every company has a role to play in making this a better society for all. […] We [each] uniquely have the power to activate in really powerful ways [by] analyzing whatever feels genuine and authentic to your brand or your product or your retail experience and matching that with a really clear ask of your consumer is really powerful and then, of course, just making sure it aligns towards your mission or your business priorities. -Company #5.*

Still, stakeholders are experiencing growing pains in the implementation of their mostly nascent approaches to achieving social impact. But, with lofty goals that can be expected:

*Yes, well, the objective is to change capitalism. That is either by supporting organizations that can raise awareness and provide information […] and frameworks to try to reform capitalism to make it more inclusive. The other part of it is making investments that are intended to generate double or triple bottom line returns: so […] single bottom line is financial, double is, financial and social, and triple is financial, social, environmental. -Investor #1.*

### Measurement today

All stakeholders want to ensure the factors they perceive to be most important are being measured. But, many interviewed perceived existing frameworks to be focused on risk, as opposed to impact, and were not sufficiently nuanced for their needs and entailed overly burdensome implementation. For investors, it is especially important to be able to tie that impact to financial benefit—as a result, several reluctantly had to create their own. Today, companies do measure impact and investors do conduct diligence and monitor investments accordingly, but for the most part, this does not touch on health outside of healthcare.

Impact reporting needs to be a distinct effort from business operations reporting—even though both contribute to financial ROI. This separation is necessary to avoid impact initiatives from being superseded by short-term cost-cutting efforts, and to drive acceptance of short-term trade-offs to accrue long-term non-financial material gains.

*It’s going to take time [to address disparities through impact investments] and I think it’s important for corporations to be realistic about that, and realistic with the folks that this work matters too, so that we all can understand, this is not a knee jerk reaction to the theme of the day. -Investor #9.*

The onus is on the company to conduct such reporting, for investors to be able to respond to any data. Quantitative data is seen as more objective here, and a perceived lack of rigor when it comes to measuring impact efforts broadly precludes growth in the impact space—especially for health.

Ultimately, metrics are an indicator of what efforts and resources are being prioritized: if you do not measure, you do not know, and you cannot compete. “Even if the number is not what it should be, […] the second you start reporting on something, the second you start tracking it, you start to improve it” (Investor #6). This matches established thought, as laid out by Nelson et al. (42), that the choice of metrics influences, organizes, and shapes behaviors early in the development of a new field or trend.

### Future of social impact

When it comes to frameworks in the future, multiple interviewees expressed that common metrics should be used as an organizing principle for determining health impact, allowing for comparison both across companies and industry verticals (e.g., education, food, commerce). This would require, and speaks to the demand for, data standardization, including success benchmarks for impact work. Improved measurement is seen as an opportunity to scale impact initiatives, and increase credibility. Many stakeholders expressed that if trustworthy health measures were readily available, there would be no obvious reason to exclude them from their impact initiatives:

*If only … capital markets could just as easily measure impact as they do financial returns … people would compete on it, and I think it would be a fascinating way for capital to find more opportunities, other than just financial aspects.*

*-Investor #4.*

Additional aspirations for future frameworks include driving equitable outcomes through product use, a requirement to report publicly to increase the accessibility of data, and a desire to capture “total returns”—that is, positive externalities that result from impact investments should be captured in the calculation of financial ROI. Indeed, it is the positive framing of externalities that could motivate companies to take action as health-conscious corporate citizens. Investors are interested in seeing their capital tied to social impact:

*I think our role as investors is demonstrating that these companies can both be designed to improve on some kind of social impact outcome and also thrive as businesses, and in fact they thrive as businesses because they are improving these social outcomes. -Investor #7.*

### Impact in the tech industry

Interviewees expressed the power of collective action, that garnering more widespread employee awareness and buy-in for impact initiatives would be a facilitator of both pace and progress. Many pointed to the need to integrate equity into product strategy to yield an equitable product development process and build product trust. Centering equity also requires the acknowledgment of both positive and negative externalities, to allow for balancing of both, and ideally being proactive to avoid the negative and to enhance the positive. Some expressed the opportunity for health impact initiatives to bridge communities on and off a product, requiring collaborative product and product policy development (among teams with agency) with external experts. There is a nervousness around first-mover disadvantage, though, where the first organization to do something might be the one that is critiqued and others can benefit from that precedent.

Still, many believed that by making health a part of their brand position, it could become a selling point:

*One thing that’s so powerful about consumers right now is that they are demanding this from the brands they consume from. Whether it’s brick and mortar retail or whether it’s a tech company, consumers now want to understand the positive role that companies are playing in society. -Company #5.*

### Motivation for considering health impact

Companies that take action to consider health impact have the opportunity for health-positive influence on market dynamics. Interviewees flagged several factors as influential toward adopting a health-promoting strategy. First, interviews suggested that externally increased regulation via government or compliance requirements would yield immediate changes. Health-promoting actions by peer companies could also create new table stakes (this was viewed as net positive, in turn acknowledging that consideration of social impact is not a zero-sum game). Investor demand could drive company differentiation or new competitive advantage. Consumers, who provide a different stakeholder perspective, are taking health more seriously, resulting in pressure on companies to adopt socially responsible practices and the need for public buy-in around their authenticity: “One thing that’s so powerful about consumers right now is that they are demanding this from the brands they consume from.” -Company #5. Internally, employees are increasingly catalyzing change around companies’ social impact decisions, both due to need for their buy-in, and their demand. Finally, modern media cycles and social media have created greater visibility and comprehension of social issues today. Of note, the latter three points were perceived to be especially resonant for Gen Z employees and consumers [consistent with Nielsen (25) and IBM (26)].

The comprehensiveness of these motivators for change indicates this is not a niche trend:

*It’s not just this warm and fuzzy thing, because we feel good about social impact, this is what your ultimate customers are thinking about and asking about, and this will help you differentiate from other products on the market. - Investor #7.*

Additional motivators for health metrics were non-stakeholder specific. Executive sponsorship could lead to company-wide accountability (indeed, CEOs account for 30% of variance around whether companies focus on corporate social responsibility) (43). The ability to influence tech policy was seen as attractive. Improved data quality, including clear attribution and digestible metrics, could convince skeptics and better capture positive product externalities. Proven cost–benefit could trigger investor or C-suite demand, and that benefit might be tied to evidence of de-risking a business or the marketability of positive impact (or of avoiding reputational risk). The rise of stakeholder capitalism could change tides. The most timely influencer, though, and which might quicken that rise, is COVID-19 (44). Companies and investors outside the healthcare sector were previously not convinced there could be “Health in All Companies,” and that health outcomes were of material value to private sector businesses. Increasingly, these entities are realizing that health is a prerequisite, both for financial participation and regular functioning. Indeed, this is an indicator that COVID-19 could change corporate priorities if the opportunity is appropriately leveraged: focusing on health and health equity in products could become an earning platform.

### Barriers to considering health impact

Despite the many motivators for organizations and investors, companies and investors identified several barriers to incorporating health impact into their corporate strategies. As opposed to motivators, the barriers to adoption are primarily internal to organizations. As described by one investor, lack of alignment with existing strategy can create roadblocks: “The farther upstream you get the more competing priorities there probably are, but I think you can make the case for … many … different kinds of verticals [vis-a-vis their health impact] at the top of the funnel” (Investor #7). Indeed, the financial margin to pivot priorities can be substantial as it is costly to stand up programs, both due to resources (financial and talent) and a question of who bears the cost (the company or consumer). The false stigma of concessionary returns does not help this belief (45). Without executive buy-in and internal collaboration, this margin is unlikely to be overcome. Part of this cost is the burden, both financial and labor, for additional reporting, which is heightened by ongoing tension debating the industry- vs. company-specific metrics, the appropriate atomic unit of measurement (e.g., company vs. product vs. algorithm), and the degree of appropriate reporting. For example, not all consumers’, or investors’, priorities are the same. Adoption by the majority would be necessary to maximize utility. Further, perceived inability to audit data according to off-product behavior, disparate data standards, and lack of direct attribution and rigor for health data creates further barriers around reporting:

*I think what’s getting in the way is finding an actual metric with confidence behind it and socializing it as something that is really essential for the whole company to think about and that, in part, is going to come from mistakes made. -Company #6.*

Further, buy-in among employees and leadership is difficult with tension around individual decisions (both of those doing the work, and those using a product) and company-level accountability for social impact decisions. The sole external barrier consistently noted was the worry around regulatory issues and possible market pushback (e.g., why is a company/investor not already doing this, and if peers have better results).

## Discussion

Social impact can be understood in multiple ways: it is a social responsibility, either by preventing harm or creating positive outcomes, within companies (i.e., through Diversity Equity, Inclusion, and Belonging initiatives), products, or in communities and/or at the intersection of the two. Social impact is increasing in importance for both corporate and investor stakeholders, but companies must apply the tactics investors are seeking in impact-driven investments. Current impact reporting demonstrates a need for “horizontal” metrics: most measures center on industry verticals, e.g., education, housing, and healthcare. Horizontal metrics allow for a sector-agnostic comparison, and a more macro-level assessment of impact, which both investors and companies yearn for as differentiators (e.g., diversity, employee satisfaction). Measuring health across verticals would be such a metric, and what would need to happen to drive a “Health in All Companies” mindset. This gap in impact frameworks highlights an opportunity through which health can play a more significant role in impact initiatives: it does not appear that there is one that stands out as gaining widespread, gold standard-level adoption.

There is a strong appetite among private sector stakeholders for considering health impact. First, the private sector needs a proof of concept, a starting point from which others can witness the path to implementation. The value and feasibility of doing so need to be socialized.

### Recommended process for bringing health into impact strategy

**Figure 3:**
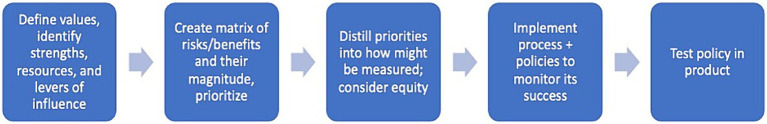
Logic model.

The process for developing a health impact strategy should be seen as the organizing principle: this is the roadmap to determine a measurement system, as is outlined in the logic model in Figure 3. Organizations must adopt a theory of change approach, where they break down the inputs, outputs, and outcomes to understand the relationship between them and how to get from one to the next. This should entail (1) defining one’s values and identifying strengths, available resources (financial, labor, and talent), and possible levers of influence, (2) establishing a matrix of both potential risks and positive contributions, alongside their magnitude, then prioritizing which are most important to address and which can be tolerated, (3) distilling these priorities into how they can be measured. For example, move from “we make games,” to “games require movement,” to “movement is good for health,” “let us determine how to track movement through our product.”

Organizations need to consider both process measures, to see ROI more quickly, and second-order effects (i.e., how does how users engage on a product influence how they behave off of it, like how options presented through a meal kit service might influence cooking choices otherwise). In determining which social outcomes they can most influence, user variation must be considered to ensure equitable distribution of outcomes. After process and policy implementation, testing must occur within the product to ensure no unintended experiences are created for any user.

While building this process, organizations must remember that they should only measure what they can control. Ultimately, there needs to be a clear line from the measures selected, to their value, the reason for their implementation, and what they will accomplish, to optimize for adoption and utility.

It will be easier, and more effective, to motivate investment in health strategies such as those proposed here when they are aligned with business priorities. If these strategies require significant investment without immediate (or negative) financial returns, companies will need to confront difficult trade-offs reconciling short-term profit goals with long-term mission. This tension is inevitable given the conflicting horizons of financial vs. social returns.

### How to design for health

Companies that aspire to integrate health impact into their social impact strategy can adopt design principles to drive health-positive product development. Investors should evaluate these same traits during diligence.

**Design for empathy**: From the outset, product teams could consider users of all backgrounds and how they might experience their product. Ideally, this might include participatory design processes (46). This can prevent unforeseen consequences for particular subsets of users. Companies may consider the implications of financial accessibility here, with discounts for the healthier choice.

**Institute accountability**: As part of any existing checks that might need to be considered before the launch of a new feature, e.g., approval from legal or compliance teams, there could be a check around the health of the user experience. By adding accountability as a company-wide process, any new feature would be subject to this consideration.

**Design proactively**: By implementing the above principles, teams can avoid post-hoc bandaids that are reactive to poor health or unanticipated behavior experienced through their products. Proactive decision making, including, for example, opportunities for education around healthier choice within a product, can negate the need for reactive, less integrated solutions (this is similar to the philosophy around medical errors) (47, 48). This may include design **prominence of health-promoting** activities (e.g., healthy foods, transit options), and **prevention** of harming behaviors (e.g., design decisions that limit sleep, going outside, physical activity).

**Feature health as reward**: If designed to foster health-positive behavior, by virtue of using the product correctly, users can inherently experience positive health outcomes (e.g., a rideshare app that defaults to pick-up/drop-off a 5-min walk from the designated location point), or teams can choose to explicitly offer in-product rewards for doing so (e.g., points in a game based around physical activity).

**Consider holistic user journey**: Teams can build a user journey that encourages healthy behavior, even if it occurs outside the product (i.e., education may occur through a product, but the actual behavior can be outside of it). Based on user behavior, teams can continuously iterate on the journey so that the most intuitive experience is the healthiest experience.

### How to measure this new approach

The interviews surfaced several areas that should be considered for measurement of health impact, and a starting point to address one of the most repeated perceived barriers. Adopting these guidelines for measures can create accountability, and can act as a signal to the market that they are prioritizing health. Indeed, while interviewees expressed a tension between product-, company-, and industry-specific measures, all acknowledged some level of consistency in measurement is needed to determine which companies’ and investors’ approaches are working, should be rewarded, and should be a model for others.

**Reach and impact**, including access, affordability, ease, and equity. This includes user participation, but also how (not just if) users are using a product. For example, does a company promote product uses that might positively influence health?

**User awareness and education** around the social impact initiatives implemented and the healthier choice being presented.

**Healthy growth**, which would require defining product health and any dose–response relationship that might exist as a threshold. This may also include the health of discourse, if applicable to the product in question. Growth may also be reflected in revenue: what proportion of sales reflect healthy options.

**Product safety**, privacy: this would entail understanding the potential for harm, including negative externalities.

**Equitable distribution of outcomes** and experience, and overall fairness: this would include measuring variation based on demographics and accessibility, and self-efficacy for healthy behaviors being fostered.

**Overall proxy for general health**: a single metric for measuring health impact was sorely wanted by many, if not most, of interviewees, as well as interest in one that might be mental health-specific. A few do exist, for example, the Mental and Physical Health Domain from Harvard’s Flourishing Index (49), however, they require survey implementation and would be self-reported, as per these sample questions:

- In general, how would you rate your physical health?
  0 = Poor, 10 = Excellent
- 4. How would you rate your overall mental health?
  0 = Poor, 10 = Excellent

### Limitations

The strong agreement between investors and tech employees interviewed can be expected due to intentional recruitment for their impact focus. By participating, interviewees aimed to advance their field and practice, introducing bias. Recommendations, being grounded in individual perceptions, hinder discerning importance and extent of generalizability. Further, the relatively small sample size, though intentionally limited due to thematic saturation, introduces the possibility that the results presented may not capture the full diversity of views within the broader population of technology executives and impact-focused investors, particularly across different geographic regions.

### Conclusion

Public health has historically flagged negative commercial determinants of health (13–16), and implemented policies accordingly (e.g., limiting sugar-sweetened beverages, restricting public smoking). There is an opportunity for the private sector to help stem the tide of chronic disease, especially in this post-COVID moment. We need to normalize assuming accountability for the potential positive health impacts of companies (32), and technology specifically—perhaps by taking a page out of the HiAP playbook (33). This research can act as a foundation on which policy can be built to guide technology development that promotes positive health behaviors at the population level. We have tried the stick, what about the carrot? More research is required to cement the financial value of considering health impact. We need to move from Congressional hearings on negative health impacts of tech companies to recommendations on what they can do differently and policies to develop products with positive externalities.

## Data Availability

The raw data supporting the conclusions of this article will be made available by the authors, without undue reservation.
